# Adaptive behavior and connectance of invasive plants mediate community composition in multilayered ecological networks

**DOI:** 10.1007/s10530-025-03601-9

**Published:** 2025-06-17

**Authors:** Yuanqi Yang, Minhua Zhang, Yu Liu, Fangliang He

**Affiliations:** 1 ECNU-Alberta Joint Laboratory for Biodiversity Study, School of Ecology and Environmental Sciences, East China Normal University, Shanghai, 200241 China; 2 Department of Renewable Resources, University of Alberta, Edmonton, AB T6G 2H1 Canada

**Keywords:** Adaptive behavior, Connectance, Ecological networks, Invasion success, Linkage rule, Network degree, Community persistence

## Abstract

Much evidence has shown that adaptive behavior can greatly modulate the dynamics of food webs, but little is known about how adaptive behaviors of invasive plant species affect community composition in multilayered networks. Following a proven network model, we constructed networks of native communities that are invaded by exotic plant species based on three linkage rules. We examined the effects of both adaptive behavior and network connectance of invasive plant species on the persistence of native species and diversity-invasion success relationship. Results showed that community persistence was mainly affected by the connectance of invasive plant species regardless of adaptive behavior. Given a fixed proportion (F_1_) of native mutualist species linked to the invasive plant species, community persistence displayed an inverse hump-shaped relationship with the increasing proportion (F_2_) of native plant species linked to the invasive plant species. Compared to the results without adaptive behavior, the adaptive behavior made most negative diversity-invasion relationship become a nonlinear U-shape at fixed proportion (F_1_). In addition, the adaptive behavior made most negative diversity-invasion relationship insignificant for some proportion (F_1_) when proportion (F_2_) was fixed. It could even reverse this relationship if the invading species was more likely to link to native species already having fewer links than those having higher links. Our results underline the importance of considering adaptive behavior and the network degree of invasive plant species for understanding the effect of invasive species on the structure and composition of ecological networks.

**Supplementary Information:**

The online version contains supplementary material available at 10.1007/s10530-025-03601-9.

## Introduction

Numerous studies have shown that invasive species have profound impacts on biodiversity and ecosystem functioning, either directly or indirectly (Albrecht et al. 2014; Cronin and Haynes 2004; Hejda et al. 2009; Hui and Richardson 2019). Specifically, invasive species could not only directly result in the decline of native species (Hejda et al. 2009; Hui and Richardson 2019; Tilman 2004) but also indirectly alter the structure of ecosystems by their effect on community dynamics (Albrecht et al. 2014; Peller and Altermatt 2024). Modeling the interactions between invasive and native species is thus necessary for understanding the impacts of invasion on local communities. Modeling exercises attempting to understand interaction networks commonly address three questions: (i) how the complexity of native networks deters biological invasions (Lurgi et al. 2014), (ii) how invasive species affect network topology and persistence of native species, and (iii) how biological traits of invasive species determine invasion outcomes (Liu et al. 2022; Lurgi et al. 2014).

Theoretical analysis shows that less connected networks are more resistant to invasions (Lurgi et al. 2014). Larger network size and greater connectance generally promote native community persistence, while nestedness and modularity have contrasting effects on invasion success in antagonistic or mutualistic bipartite networks (Liu et al. 2022). Research also shows that invasion success is strongly mediated by body size and diet breadth, with larger and more generalist species being more successful at invasion (Lurgi et al. 2014). However, these theoretical studies on invasion are mostly focused on the effect of network complexity (e.g., species richness and connectance) or network properties (e.g., nestedness and modularity) on native community persistence in antagonistic or mutualistic networks. The network used in these theoretical studies only considers one type of interspecific interaction (e.g., predation or mutualism) in addition to competition at a time (Hui and Richardson 2019; Liu et al. 2022; Lurgi et al. 2014).

In reality, natural communities are complex assemblages of species which engage in various intraspecific and interspecific interactions (e.g., competition, mutualism, predation and parasitism). For example, flowering plants can benefit pollinators while they are consumed by herbivores and compete with other plants for nutrients, creating a complex network (García-Callejas et al. 2018a, b). This pollinator-plant–herbivore tripartite network can be considered a composite of two subnetworks: a mutualistic subnetwork (a plant-pollinator network) and an antagonistic subnetwork (a plant–herbivore network). Such a multilayered network represents a real system in nature and is more insightful in understanding the effects of complex interactions on community structure and dynamics. For example, compared with a single bipartite network, Sauve et al. (2014) found that the effects of nestedness and modularity are significantly weakened in multilayered networks composing of mutualistic and antagonistic interactions. Hence, compared with invasion in a single interaction network, multilayered interactions of invasive species are needed to understand the real impact of invasive species on native communities. Another major concern in invasion studies is how the diversity of native species affects the invasion outcome. For example, the "diversity-invasibility hypothesis" states that the higher the diversity in a local plant community (only including competitive interaction), the lower the probability of successful invasion by alien plants. However, this relationship is not invariable, which may be driven by different factors (Feng et al. 2018; Zhang et al. 2020; Zhu et al. 2015; Peng et al. 2019). It is thus necessary to explore the relationship between diversity and invasibility in multiple interaction networks.

Many studies have shown that predator and pollinator species often switch their interactive partners over time in natural communities (Fortuna and Bascompte 2006; Olesen et al. 2008; Petanidou et al. 2008; Staniczenko et al. 2010). This is called an adaptive behavior because a shift in interactive partners is often considered as a response to a changing environment. The “environment” here could mean the change in interactive partners (e.g. omnivores can shift their prey species depending on the prey abundance at the time) or the abiotic environment (e.g. resource availability and environmental disturbances) (Petanidou et al. 2008; Staniczenko et al. 2010; Whittall and Hodges 2007; Zhang et al. 2011). Plants can also show adaptive behavior to attract pollinators, including the modification of their flowering phenology, nectar amount and the chemical composition of their nectar or secretions (Ågren 2019; Trunschke et al. 2024; McKinney and Goodell 2011). Compared with fixed interactions between species in food webs, models considering adaptive foraging of consumer species (i.e., changing interaction strength) could provide theoretical insights into why real ecosystems often support a large number of interacting species (Beckerman et al. 2006; Garcia-Domingo and Saldaña 2007; Kondoh 2003, 2006; Petanidou et al. 2008; Staniczenko et al. 2010). In plant–pollinator mutualistic networks, models considering the switch of pollinator’s partners can predict the nested architecture of empirical networks, suggesting interaction switch is an important ecological process (Kaiser-Bunbury et al. 2010; Zhang et al. 2011). Studies also show that adaptive behavior is a mechanism critical to the stabilization of networks (Bartley et al. 2019; Garcia-Domingo and Saldaña 2007; Kondoh 2003, 2006; Mougi 2016, 2022; Valdovinos et al. 2016, 2010). Therefore, when studying species invasion, it is necessary to investigate the effect of adaptive behavior (e.g. the change in interaction strength between an invasive species and different native mutualist species) on native community persistence, structure and stability of invaded networks.

In this study, we construct a mathematical model to describe invasion dynamics in a mutualist-plant–herbivore multilayered network. First, we start with Yan’s (2022) original network model which does not consider plant invasion and assumes the network is stable, i.e., the native species coexist stably. We then introduce an invasive plant species to that model consisting of both antagonistic and mutualistic subnetworks where herbivores and pollinators interact with a shared group of native plants. We finally investigate the effect of adaptive behavior of invasive plant species on the native community persistence under different normalized link degrees and linkage rules. Here, “degree” is defined as the number of links between an invasive species and other species in a network (including interactions with native plants, pollinators and herbivores) so that normalized link degree is a measure of network connectance. The invasive plant species can select either the high-degree or low-degree native species or randomly selects any native species of an interested guild. These different ways of selection are called linkage rule. Our objective is to assess the effect of adaptive behavior of invasive species on the persistence of native community and establish relationships between native diversity and probability of successful invasion under different normalized link degrees and linkage rules of invasive species. Our results show that network structure combined with adaptive behavior has profound effects on native community persistence and the relationship between native diversity and probability of successful invasion.

## Material and methods

### Study system

Many models have been developed to study ecological networks, e.g., cascade models, niche models and bipartite models (Chen and Cohen 2001; Thébault and Fontaine 2010; Williams and Martinez 2000). Most of these models focus on modeling food webs (which describes a classical representation of “who eats whom” in a community) or bipartite networks (in which no interactions within the same trophic level are assumed). In a recent study on the effect of nested topologies on ecosystem stability, Yan (2022) constructed a multilayered network model consisting of plant-mutualist and plant–herbivore subnetworks. These two subnetworks are connected through shared plants, forming a multilayered network that includes three guilds (plants *S*_*p*_, mutualists *S*_*m*_ and herbivores *S*_*h*_). Species in the network involve three types of interactions: competitive, mutualistic and antagonistic. In this study, we extend this multilayered network to construct a model that considers plant invasion. We start with this network model without considering plant invasion, i.e., Yan’s (2022) original model, and require the network be stable, i.e., the native species coexist stably. We then proceed to analyze the model by including plant invasion.

When an invasive plant species is introduced into the stable network, we denote ${F}_{1}$ as the proportion of native mutualist species (e.g., pollinators) linked to the invading plant species and ${F}_{2}$ as the proportion of native plant species (e.g., competitors) linked to the invading species, where both $F$’s fall in [0, 1]. ${F}_{1}$ is calculated as the link degree of invasive plant species divided by the number of species in native mutualists guild. ${F}_{2}$ is calculated as the link degree of invasive plant species divided by the number of species in native plants guild of interest. ${F}_{1}$ and ${F}_{2}$ are called normalized degree, which re-scales link degree (Dormann 2011). Different ${F}_{1}$ and ${F}_{2}$ values represent different magnitudes of normalized link degree between the native community and the invasive plant species. The normalized degree of invasive plant species was initially set by $F=$ 0 to 1 with increasing intervals 0.05, following Liu (2022) and Wang (2023). High *F* values indicate high normalized degree (i.e., more potential interactions between the invasive and native species). Based on these proportions, a specified number of native species is assigned to interact with the invading species (here, a specified number equals the normalized degree multiplied by the species richness of the native guild of interest). Interaction links between the invading species and the native species are selected following three rules: (a) Random linkage – randomly selecting native species irrespective of their link degree. This random linkage means that the invading species can compete with any native plant species or be pollinated by any native mutualist species with equal probability; (b) Most-to-Least linkage – species interactions are selected in the decreasing order of their linking degree, i.e. starting from native species with the most links to the least links; and (c) Least-to-Most linkage – species interaction links are selected in the increasing order of their linking degree, i.e. starting from species with least links to the most links (Liu et al. 2022; Wang et al. 2023). Under the Least-to-Most rule, competition with native plants for the same pollinator can be avoided if invasive plants are more inclined to select pollinator species that have less interaction with native plants. However, under the Most-to-Least rule, high-degree mutualist species have many plant species sources. The preference of invasive plants for generalist pollinators species means that there are no pollination restrictions. For example, if the invasive plant possesses characteristics that strongly attract generalist pollinator species, such as flower colors favored by these pollinators, it can be easily visited by generalist pollinators (Most-to-Least) (Stout 2007; Powell et al. 2011). Studies have shown that although herbivores preferentially feed on native plants, some native herbivore species also feed on invasive plant species (Guyton et al. 2020; Zhang et al. 2018). In this study, we assume that native herbivore species feed on invasive plant species with probability $P=0.6$.

### Community dynamic model

We extend the model of Yan (2022) to construct the following model with plant invasion in a multilayered community with a varying proportion of interaction links between native and invasive species. We explore the effects of adaptive behavior (reallocation of interaction strength between invasive plant species and native mutualist species) and the normalized degree of invasive plant species on community composition (Albrecht et al. 2014; Liu et al. 2022; Yan 2022). This leads to the multilayered community model:

1 $$\frac{{dP_{i} }}{dt} = P_{i} \left( {r_{{P_{i} }} - d_{{P_{i} }} P_{i} - \sum\limits_{j = 1}^{{S_{P} }} {\alpha_{ij}^{pp} P_{j} } - \phi_{k} \alpha_{ik}^{pp} P_{k} + \sum\limits_{l = 1}^{{S_{M} }} {\alpha_{il}^{pm} M_{l} } - \sum\limits_{h = 1}^{{S_{H} }} {\alpha_{ih}^{ph} H_{h} } } \right)$$

2 $$\frac{{dM_{l} }}{dt} = M_{l} \left( {r_{{M_{l} }} - d_{{M_{l} }} M_{l} - \sum\limits_{y = 1}^{{S_{M} }} {\alpha_{ly}^{mm} M_{y} } + \sum\limits_{i = 1}^{{S_{P} }} {\alpha_{li}^{pm} P_{i} } + \phi_{k}^{\prime } \alpha_{lk}^{pm} P_{k} } \right)$$

3 $$\frac{{dH_{h} }}{dt} = H_{h} \left( {r_{{H_{h} }} - d_{{H_{h} }} H_{h} - \sum\limits_{r = 1}^{{S_{H} }} {\alpha_{hr}^{hh} H_{r} } + \sum\limits_{i = 1}^{{S_{P} }} {\alpha_{hi}^{ph} P_{i} } + \varphi_{k} \alpha_{hk}^{ph} P_{k} } \right)$$

where *P*, *M*, and *H* represent the population density of plants (including the invasive plant), mutualists and herbivores, respectively. We designate *i* and *j* to represent native plant species, *l* and *y* to represent native mutualistic species, *h* and *r* native herbivore species and *k* invasive plant species. In addition, *P*_*k*_ is the population density of invasive plant species *k*. In each simulation iteration, initial native and invasive species abundances are randomly selected from a uniform distribution $U[0.1, 10]$ and$U[0.1, 2]$, separately. $r_{{P_{i} }}$, $r_{{M_{l} }}$ and $r_{{H_{h} }}$ are the intrinsic reproduction rates of species from guild *i*, *l* and *h*, randomly sampled from$U[0.5, 1.5]$,$U[-0.5, 0.5]$, and$U[-1, 0]$, respectively. These uniform distributions assume that mutualists could be facultative or obligate, and herbivores rely on plants to survive. ${d}_{x}$ ($x = P_{i} ,M_{l} ,H_{h}$) is the intraspecific density-dependent term (randomly sampled from $U[0.5, 1.5]$ for introducing variation to negative density-dependent effects). $\alpha_{zq}^{xx}$ is the competition coefficient between species *i* and *j* from same guild ($xx=pp,mm,hh$;$zq=ij,ly,hr$); $\alpha_{ih}^{ph} (\alpha_{il}^{pm} )$ is the predation (mutualism) coefficient of species *h* on *i* ($a_{il}^{pm} = 0$ when species *i* does not interact with species *l*). $\alpha_{zq}^{xx}$, $\alpha_{ih}^{ph} (\alpha_{hi}^{ph} )$ and $\alpha_{il}^{pm} (\alpha_{li}^{pm} )$ are independently drawn from half-normal distributions $|\text{N}\left[0, {\sigma }^{2}\right]|$ with a mean = 0 and a standard deviation ($\upsigma$) drawn from$\text{U}[\text{0.05,0.2}]$. But we assume standard deviation of intraguild competition for mutualist and herbivore lower than that for native plants ($\upsigma \sim \text{U}[\text{0.03,0.05}]$). ${\phi }_{k}=1$ represents that native plant species can interact with invasive plant species or otherwise not (i.e.${\phi }_{k}=0$). Similarly, ${\phi }_{k}{\prime}=1$ indicates that there exists an interaction link between native mutualist species and invasive plant species or otherwise not (i.e.${\phi }_{k}{\prime}=0$). $\varphi_{k} = 1$ indicates that there exists an interaction link between native herbivore species and invasive plant species or otherwise not (i.e. $\varphi_{k} = 0$).

In the presence of adaptive behavior, the observed connection probability can be smaller than the potential normalised degree as some links between invasive plant species and native mutualist species could disappear. The population dynamics and the change in interaction strength (the adaptive behavior) of invasive plant *k* to mutualist species *i* ($\alpha_{ki}^{pm}$) are given by:

4 $$\frac{{dP_{k} }}{dt} = P_{k} \left( {r_{{P_{k} }} - d_{{P_{k} }} P_{k} - \sum\limits_{i = 1}^{{S_{P} }} {\alpha_{ki}^{pp} P_{i} } + \sum\limits_{l = 1}^{{S_{M} }} {\alpha_{kl}^{pm} M_{l} } - \sum\limits_{h = 1}^{{S_{H} }} {\alpha_{kh}^{ph} H_{h} } } \right)$$

5 $$\frac{{d\alpha_{kl}^{pm} }}{dt} = G_{k} \alpha_{kl}^{pm} \left( {\frac{{\partial W_{kl} }}{{\partial \alpha_{kl}^{pm} }} - \sum\limits_{l \in mutualists} {\alpha_{kl}^{pm} \frac{{\partial W_{kl} }}{{\partial \alpha_{kl}^{pm} }}} } \right)$$

$r_{{P_{k} }}$, $d_{{P_{k} }}$ and $P_{k}$ are chosen from$U[0.5, 1.5]$, $U[0.5, 1.5]$ and$U[0.1, 2]$, respectively. $\alpha_{ki}^{pp}$ and $\alpha_{ik}^{pp}$ are the competition coefficient between invasive plant species *k* and native plant species *i*; $\alpha_{kl}^{pm}$ and $\alpha_{kh}^{ph}$ are the mutualism and predation coefficient of mutualist species *l* and herbivore species* h* on *k*. They are independently drawn from half-normal distributions $|\text{N}\left[0, {\sigma }^{2}\right]|$ with the standard deviation ($\upsigma$) drawn from$U[0.3, 0.5]$,$U[5, 5.5]$, $U[0.05, 0.2]$ and$U[0.05, 0.2]$, respectively. Here, standard deviation for the competition coefficients ($\alpha_{ki}^{pp}$ and $\alpha_{ik}^{pp}$) between invasive plant species and native plant species is not sampled from the same uniform distribution. This makes it more likely that invasive plant species be more competitive than native plant species (see Supplementary Materials for the results which assume the standard deviations for $\alpha_{ki}^{pp}$ and $\alpha_{ki}^{pp}$ are from the same uniform). We further set that the mutualism and predation coefficient generation methods of mutualist species *l* and herbivore species* h* on *k* are assumed to be the same as mutualist species *l* and herbivore species to native plants. ${W}_{ki}$ is the per-capita growth rate (${dR}_{k}/{R}_{k}dt$) of the competitors or mutualists and ${G}_{k}$ is the adaptation rate of invasive plant species. ${G}_{k}=0$ represents invasive plant species has no adaptive behavior. In other words, the strength of interactions does not change over time for invasive species without adaptive behavior (Mougi 2016, 2022). Moreover, we assume that the total amount of interaction strength in the presence of adaptive behavior equals that in the absence of adaptive behavior. For simplicity, we further assume adaptation rate is a constant to all species (${G}_{k}=0.5$) when considering adaptive behavior.

### Analysis of community properties

The complexity of the above model prevents an analytical evaluation of the model. We thus numerically analyze the model using ode 45 in Matlab R2019b. We run the dynamic system for a sufficiently long time ($t=2\times {10}^{3}$, which corresponds to the time a species takes to reach an asymptotic steady state) and then record relevant matrices such as population size and species interactions (in which the element ${a}_{ij}$ represents the interaction strength between species $i$ and species $j$) after the system reaching equilibrium. During each iteration, we define species extinction if its population size falls below a threshold of 10^–6^ and successful invasion if population size of invasive plant species is above this threshold. As an index of community stability, community persistence is calculated as the percentage of species that remain above this 10^–6^ threshold at the end of a simulation over the initial species richness of the network (excluding invasive plant species). This community stability is calculated for various scenarios that assume different proportions (F_1_) of native mutualist species linked to the invasive plant species and different proportions (F_2_) of native plant species linked to the invasive plant species (${F}_{1}$,${F}_{2}$) and linkage rules in this study (Kondoh 2003; Mougi 2016). As some studies have shown that native diversity play an important role in resisting invasion, we use the generalized additive models (GAMs) to model relationships between native diversity and probability of successful invasion using the gam function in the R package ‘mgcv’ (Wood 2017). Hereafter, native diversity refers to the total species richness of the three native guilds.

## Results

### The role of different normalised degrees of invasive plant species and linkage rules in community persistence

**Fig. 1:**
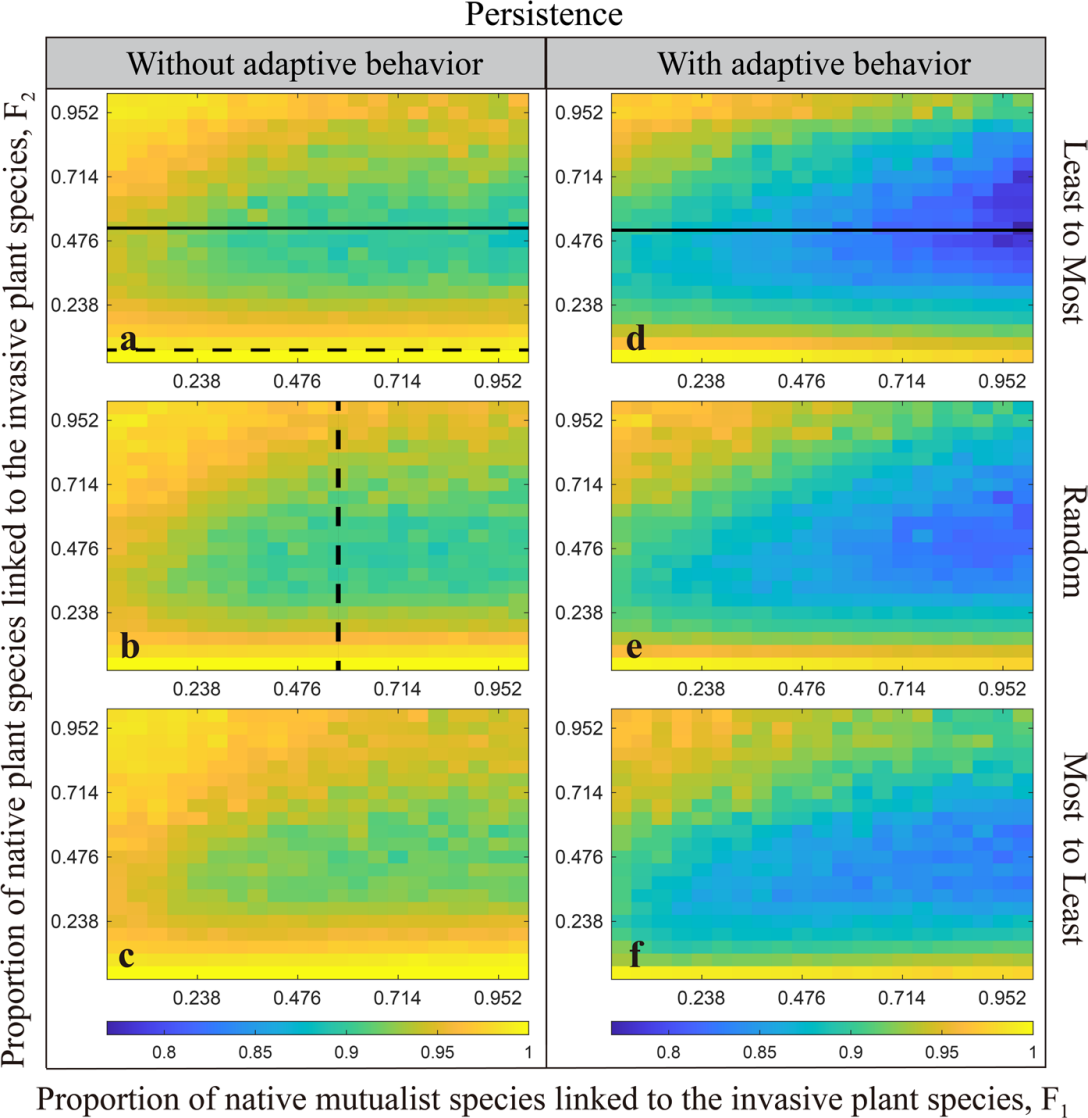
Mean community persistence at steady state (averaged over 4 replicates) after an invasion to mutualist-plant–herbivore multilayered networks. The proportions (${F}_{1}$ or ${F}_{2}$) of native (mutualist or plant) species linked to the invader are varied ($0\le {F}_{1}, {F}_{2}\le 1$). Three linkage rules for invader linking to native (both mutualist and plant) species are considered: Least-to-Most (**a** and **d**), Random (**b** and **e**) and Most-to-Least (**c** and **f**). **a**, **b** and **c** show invasive plant species without adaptive behavior. **d**, **e** and **f** are the scenarios with adaptive behavior. Colors represent the mean community persistence. Note community persistence refers to the native species (including all guilds), not including the invader

Results in Fig. 1 show that the community persistence of native species (including all guilds) is decreased dramatically with increasing proportion (F_1_) of native mutualist species linked to the invasive plant species, and at a moderate proportion (F_2_) of native plant species linked to the invasive species, irrespective of linkage rules or adaptive behavior (solid black line in Fig. 1a and 1d). Furthermore, community persistence is not affected at a low proportion (F_2_) of native plant species linked to the invasive plant species (dashed black line in Fig. 1a and the change can also be seen in Fig. S2 in supplementary materials). However, the overall persistence of the community displays an inverse hump-shape with increasing proportion of competitors (F_2_) at a fixed proportion of mutualists (F_1_) (dashed black line in Fig. 1b). In contrast, the presence of adaptive behavior can enhance the influence of the proportion of interaction links between invasive plant species and native species on community persistence (e.g., Fig. 1a versus d).

### The effects of adaptive behavior and normalized degree of the invasive plant species on the relationship between native diversity and invasion success

**Fig. 2:**
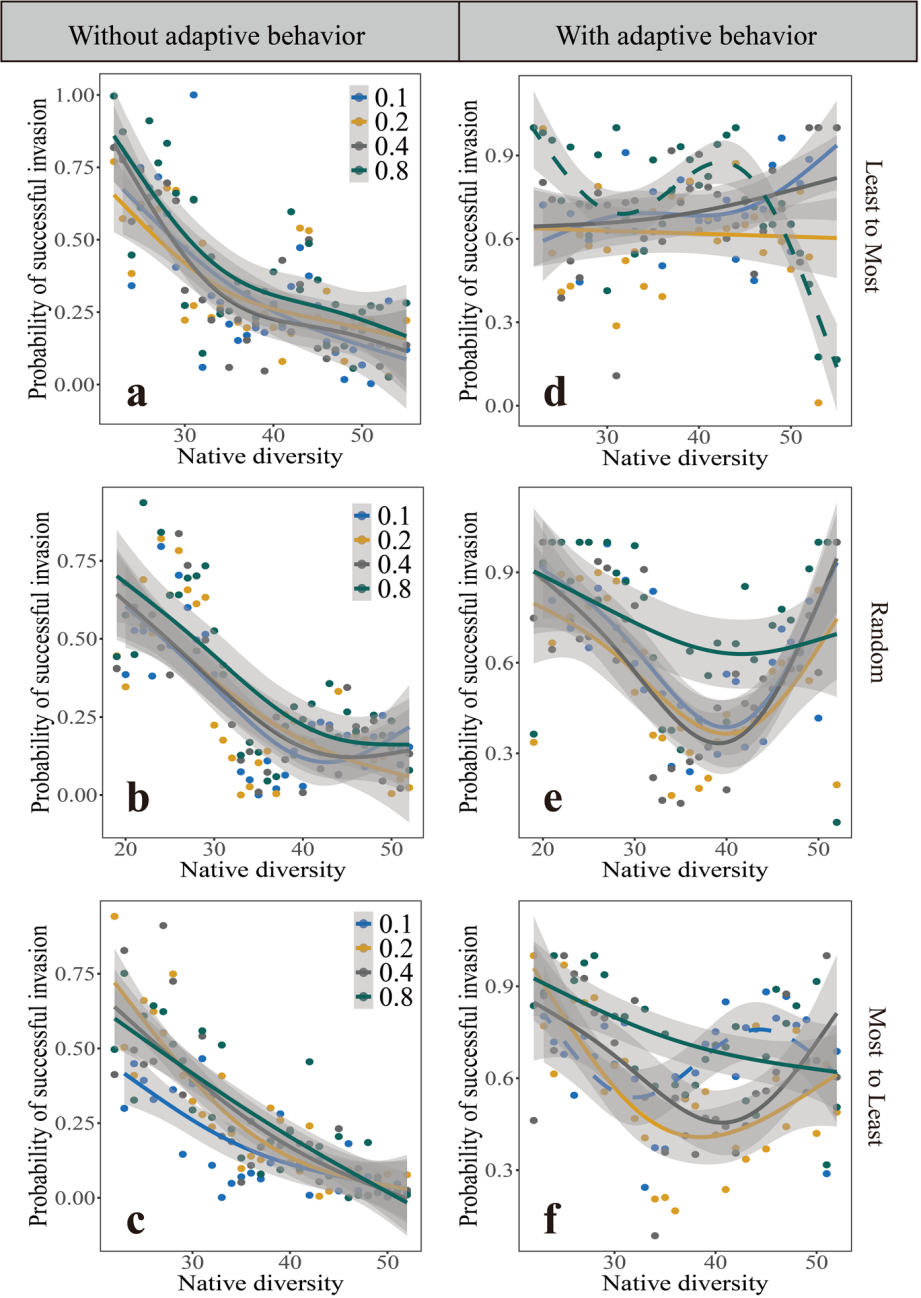
The relationships between native diversity and the probability of successful invasion, evaluated at different proportions (${F}_{2}$) of native plant competitors linked to the invasive plant species. Each column from top to bottom corresponds to Least-to-Most (a and d), Random (**b** and **e**) and Most-to-Least linkage rules (**c** and **f**), respectively. **a**, **b** and **c** are invasive plant species without adaptive behavior and **d**, **e** and **f** are with adaptive behavior. Colors indicate different proportions (${F}_{2}$) of native plant species (${F}_{2}=0.1, 0.2, 0.4, 0.8$) linked to the invasive plant species at a fixed proportion (${F}_{1}$) of native mutualist species linked to the invasive plant species (${F}_{1}=0.4$). Dashed curves indicate nonsignificant regressions, while solid lines are significant at $P<0.05$. Native diversity refers to the total species richness of all native guilds, not including the invader

The results in Fig. 2 show the effects of adaptive behavior and the magnitude of normalized degree on the relationship between native diversity and the probability of successful invasion. First, we fixed ${F}_{1}$ (proportion of native mutualist species linked to the invasive plant species) while changing ${F}_{2}$ (proportion of native plant competitors linked to the invasive plant species). In the absence of adaptive behavior, there is a consistently significant negative relationship between native diversity and the probability of successful invasion, regardless of the linkage rules (Fig. 2a, b and c). However, with adaptive behavior, this relationship is inconsistent across linkage rules (Fig. 2d, e and f). For example, at a low or intermediate proportion (${F}_{2}$) of native plant competitors, a positive relationship between invasion success and native diversity is observed under the linkage rules of Least-to-Most (Fig. 2d). But at a moderate proportion of ${F}_{2}$ (= 0.2, 0.4), the probability of successful invasion and native diversity show a U-shape relationship under the linkage rules of Random and Most-to-Least (Fig. 2e and f). These results indicate that, when the proportion (${F}_{1}$) is fixed, the adaptive behavior could change the strong negative relationship between native diversity and successful invasion into a positive or nonlinear U-shaped relationship (under moderate proportion (${F}_{2}$) of native plant competitors).

**Fig. 3:**
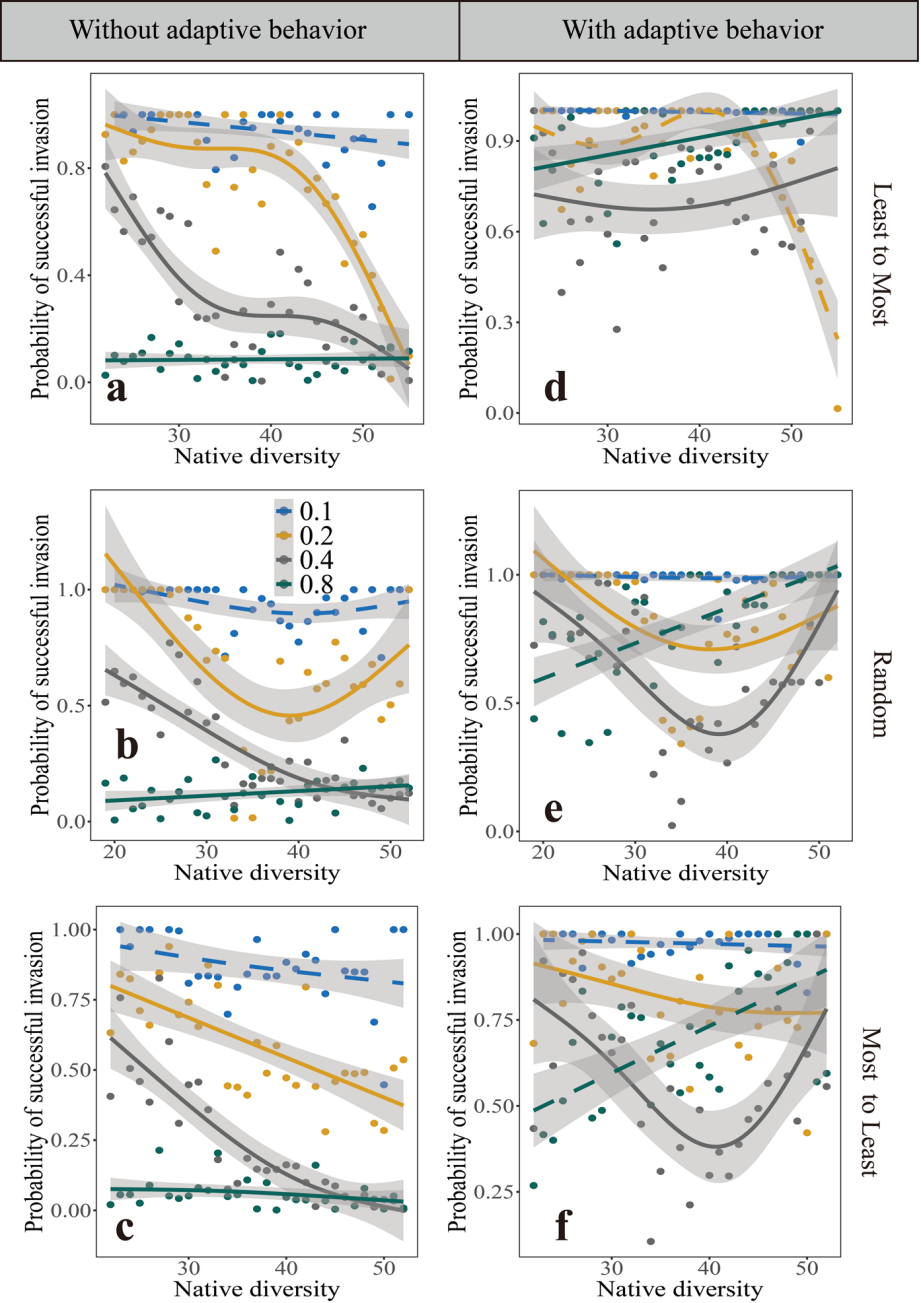
The relationships between native diversity and the probability of successful invasion, evaluated at different proportions (${F}_{1}$) of native mutualist species linked to the invasive plant species. Column panels from top to bottom corresponds to Least-to-Most (**a** and **d**), Random (**b** and **e**) and Most-to-Least linkage rules (**c** and **f**), respectively. **a**, **b** and **c** are invasive plant species without adaptive behavior and **d**, **e** and **f** are with adaptive behavior. Colors indicate different proportions (${F}_{1}=0.1, 0.2, 0.4, 0.8$) of native mutualist species linked to the invasive plant species at a fixed value (${F}_{2}=0.4$). Dashed lines indicate nonsignificant relationship at $P<0.05$. Native diversity refers to the total species richness of native plants, pollinators, and herbivores

We next fixed ${F}_{2}$ (proportion of linked native competitors) while changing ${F}_{1}$ (the proportion of linked native mutualists). Results in Fig. 3 show the effect of normalized link degree with adaptive behavior on the relationship between native diversity and the probability of successful invasion. Under a fixed proportion (${F}_{2}$) of native plant competitors, adaptive behavior makes the relationship between native diversity and the probability of successful invasion nonsignificant for some proportions of interaction links and regardless of the linkage rules (Fig. 3b versus e; c versus f). A high proportion (${F}_{1}$) of native mutualist species (${F}_{1}$=0.8) shows a significant positive relationship is maintained when the linkage rule is Least-to-Most (green solid line in Fig. 3d). Therefore, positive relationships may occur regardless of which values of (${F}_{1}$) native mutualist species and (${F}_{2}$) native competitor species are fixed. Compared to the changes in Fig. 3d, the adaptive behavior of the invasive species could result in U-shape relationships under moderate proportion (${F}_{2}$) of native plant species (Fig. 3e, f). According to results in Fig. 2 and Fig. 3, the positive relationship between successful invasion and native diversity is more likely to appear when the linkage rule is Least to Most, but the nonlinear U-shape is more likely to occur when the linkage rules are random and Most to Least.

## Discussion

The invasion of alien species is one of the most significant threats to biodiversity worldwide (Peller and Altermatt 2024; Pyšek and Richardson 2010; Ricciardi et al. 2017; Simberloff et al. 2013). To predict the impact of invasive species on native communities, it is necessary to understand how the magnitude of interactions between invading and local species, the evolution of the interactions, and the diversity of the local community affect species composition. In this study, we extend a multilayered network model (Yan 2022) to model the effect of an invasive plant species on networks composing of native plants, mutualists and herbivores. We analyze the effects of adaptive behavior (reallocation of interaction strength between invasive plant species and native mutualist species) and normalized link degree with native species on the success of species invasion and the persistence of native species. Regardless of adaptive behavior, we show that community persistence is not affected by the initial interaction link selection between invasive and native species. In other words, changes in community persistence are consistent regardless of whether the invasive plant species interacts with the high-degree or low-degree native species or randomly interacts with native species. This is consistent with previous studies for bipartite networks that show community persistence is independent of linkage rules but it follows that invasive species would exclude less competitive native plants, allowing for secondary extinctions of specialist pollinators and consumers that interact with these native plants (Liu et al. 2022).

Linkage rules have significant effects on the relationship between native diversity and probability of successful invasion compared to the scenario of no adaptive behavior. With adaptive behavior, the invasive plant interacting with low-degree mutualist species could reverse the negative relationship into positive one. This means that the higher native diversity, the more likely the invasive plant would be successful invasion. But under Random and Most-to-least rules, U-shape relationship is more likely to occur at intermediate proportions (${F}_{1}$ or ${F}_{2}$) (Fig. 2e, f and Fig. 3e, f). These suggest that the adaptive behavior and linkage rules of invasive species are important in affecting the establishment of invasive species, the biodiversity of the invaded communities and the relationship between native diversity and probability of successful invasion.

Our model shows, over a wide range of normalized link degrees of invasive plant species (i.e., network connectance), linkage rules do not strongly constrain species coexistence (Fig. 1). The linkage rules just weakly increase community persistence in the order of Least-to-Most < Random < Most-to-Least (Fig. 1). This result is consistent with the findings that in food webs (varying from minimally constrained random networks to highly structured networks; Williams 2008) and host-parasite networks (Su and Yang 2020), initial network structure only weakly affects community persistence. When increasing the proportion (${F}_{1}$) of native mutualist species linked to the invasive plant species, at a fixed low proportion (${F}_{2}$) of native plant competitions, native community persistence is found to decrease (solid black line in Fig. 1a). This can be explained because increasing the number of links between invasive plant and mutualist species will enable invasive species to derive greater benefits from mutualisms (also see Albrecht et al. 2014; Memmott and Waser 2002; Richardson et al. 2000). This rise in invasive species abundance can, in turn, heighten the risk of extinction for less competitive native plant species, thereby diminishing the persistence of native plants via competitive exclusion.

We find the inverse hump-shape of community persistence with increasing proportion (${F}_{2}$) of native plant competitors (at a fixed proportion (${F}_{1}$) of native mutualist species) (dashed black line in Fig. 1b) is interesting. This shape could result from the following process. If the number of native mutualist species linked to invasive plant species (${F}_{1}$) is fixed, native community persistence is mostly determined by the number of competitive links between invasive and native plant species. Therefore, invasive plant species may drive extinction of the less competitive native plants when the magnitude of normalized link degree is at a low level (i.e., few competitive interactions) (Wu et al. 2024). However, when the magnitude of normalized link degree increases to a certain level, the competition strength of some native plant species could be greater than that of invasive species and these native species will lead to a competitive disadvantage of invasive species and limit the abundance of invasive plant species. Correspondingly, native pollinator species can have more opportunities to pollinate with less competitive native plants, which will increase the survival probability of these native plants. As a result, these native plant species that are less affected by invasive plants will survive and community persistence will increase.

An important result of our study is that adaptive behavior could change the relationship between native diversity and probability of successful invasion (Fig. 2 and 3). Regardless of linkage rules, in the absence of adaptive behavior, there is a significant negative correlation between native diversity and probability of successful invasion at a given proportion (${F}_{1}$) of native mutualist species (Fig. 2a, b and c ). This could be explained by the fact that communities with high species diversity have fewer spare niches, leaving less resources for alien plants to exploit, thus reducing their chances of invasion (Elton 1958; Case 1990; Levine 2000; Zheng et al. 2018). When fixing the proportion (${F}_{2}$) of native plant competitors, but at an intermediate proportion (${F}_{1}$) of native mutualists, there is almost a negative correlation between native diversity and probability of successful invasion (yellow line and brown in Fig. 3a, b, c). The reason for this is that with the increasing proportion (${F}_{1}$) of native mutualist species, native mutualists favor the more abundant native plant species, thereby providing a mechanism of majority-advantage that potentially inhibits the invasive plant species (Loeuille 2010).

On the contrary, in the presence of adaptive behavior, a positive relationship results at a high proportion of native mutualist species linked to the invasive plant species (${F}_{1}=0.4, 0.8$) when the linkage rule is Least (Fig. 3d). This may be because the low-degree mutualist species only have a few plant sources and the adaptative behavior of invasive plant species can attract those abundant mutualist species, resulting in higher invasion rates. With adaptive behavior, the U-shape relationship between native community diversity and successful invasion is more likely to occur at intermediate proportions when the linkage rules are Random or Most-to-Least (Fig. 2e, f and Fig. 3e, f). The reasons could be as follows: Under Random or Most-to-Least rule, the probability of drawing a generalist pollinator is increased. When a community has low diversity, very few native plant species compete with invasive plant, and the adaptive behavior could make the invasive species benefit more from high-degree native pollinators, resulting in a high probability of successful invasion. As native diversity increases, the benefits of adaptive behavior may be lower than the effects of competitive interactions between native plants and the invasive plant. Native plant species defend themselves against invasive plant species, thus lowering successful invasion. However, in communities with high diversity, invasive plant species could face high niche overlap with native plant species. Previous study found that native plants that interact with high-degree pollinators are more likely to go extinct when alien plants invade (Wang et al. 2023). If some native plants that interacted with high-degree pollinators go extinct, the loss of these native plant species frees up ecological niches which would further facilitate plant invasion (Tilman 1997; Fridley et al. 2007).

In conclusion, models considering the adaptive behavior of invasive plant species in the multilayered networks show that adaptive behavior could play an important role in regulating diversity-invasibility relationships. It could change a negative relationship to a positive relationship (when links are Least to Most) or make the relationship a U-shape at moderate normalized link degree (Random and Most to Least links). We also find that the magnitude of normalized link degree, or the connectance of invasive plant species, has a great effect on native community persistence regardless of whether the invasive plant species has adaptive behavior or not. However, real ecosystems are complex, and our study is limited to the networks where the strength of interactions between invasive plant species and native mutualists changes over time, while the competitive ability of native plants species (sharing native mutualist species with invasive plant species) and the interaction strength of native mutualists and herbivores are unchanged. It has been shown that plant species sharing pollinators may compete for pollination (called pollination-mediated competition) (Aizen and Vázquez 2006). We suggest the next step of research is to investigate how adaptive behavior of native species affects community persistence and the diversity-invasibility relationship.

## Data availability statement

Codes are available on request.

## Supplementary Information

Below is the link to the electronic supplementary material.

Supplementary file1 (TIF 4411 kb)

Supplementary file2 (TIF 75638 kb)

Supplementary file3 (TIF 112208 kb)

Supplementary file4 (TIF 80706 kb)

Supplementary file5 (TIF 79698 kb)

Supplementary file6 (DOCX 1764 kb)
