# Supplementary figures and images for: Adaptive behavior and connectance of invasive plants mediate community composition in multilayered ecological networks
Source: Biol Invasions. 2025 Jun 17;27(7):156. doi: 10.1007/s10530-025-03601-9 (PMC12174227; doi:10.1007/s10530-025-03601-9)

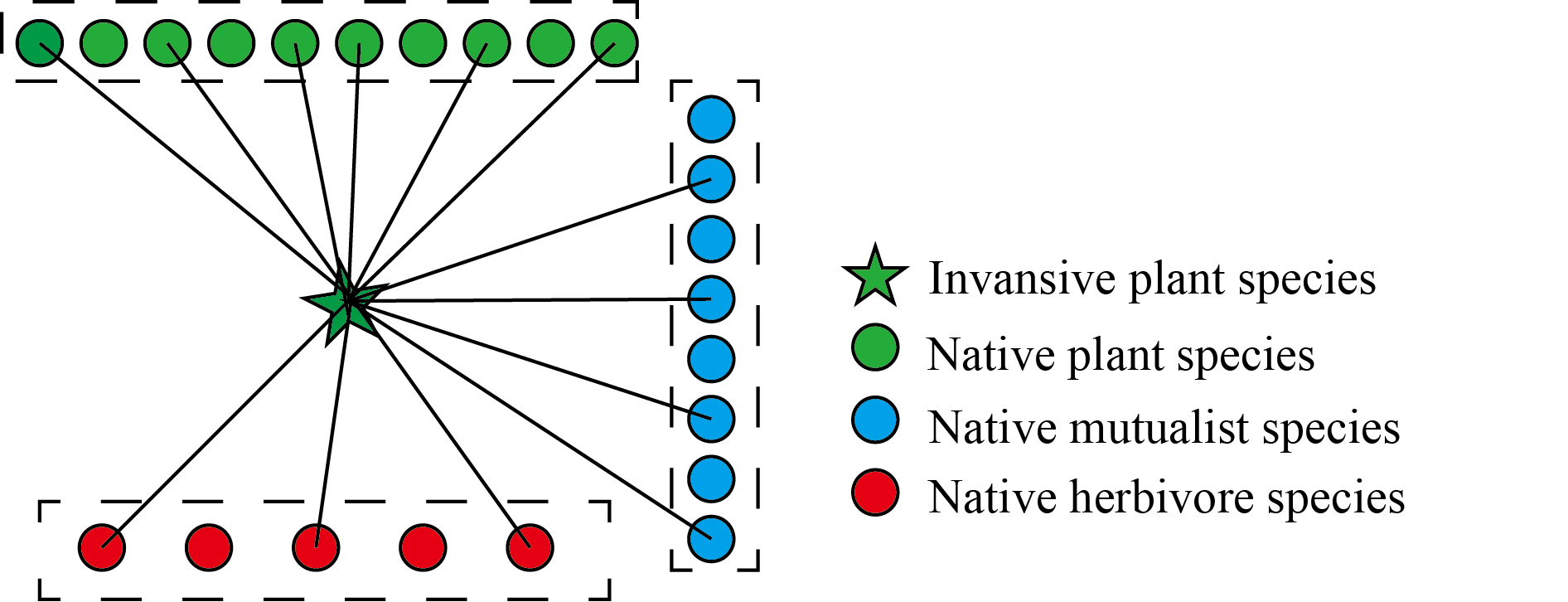

Supplement: Supplementary file 1 — Supplementary file1 (TIF 4411 kb) [file 10530_2025_3601_MOESM1_ESM.tif]
