# Supplementary material for: Adaptive behavior and connectance of invasive plants mediate community composition in multilayered ecological networks
Source: Biol Invasions. 2025 Jun 17;27(7):156. doi: 10.1007/s10530-025-03601-9 (PMC12174227; doi:10.1007/s10530-025-03601-9)
Supplement: Supplementary file 6 — Supplementary file6 (DOCX 1764 kb) [file 10530_2025_3601_MOESM6_ESM.docx]

**Supplementary Materials**

**Adaptive behavior and connectance of invasive plants mediate community composition in multilayered ecological networks**

Yuanqi Yang, Minhua Zhang, Yu Liu, Fangliang He

**
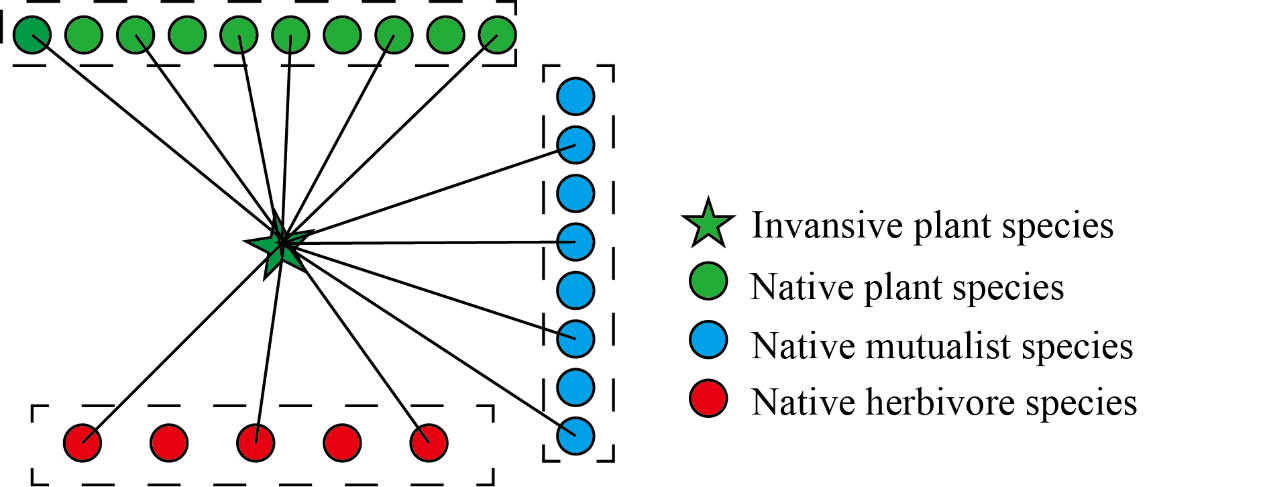
**

**Fig. S1.** Schematic diagram showing the interactions between invasive plant species and native species. Pentagram node is invasive plant species and dot nodes are native species (green: native plant species; blue: native mutualist species; and red: native herbivore species). Links show interaction between invasive and native species. In the main text, we donate $F_{1}$ as the proportion of native mutualist species linked to the invading plant species and $F_{2}$ as the proportion of native plant species linked to the invading species. As an example of calculation for $F_{1}$ and $F_{2}$, there are four links between the invasive species and the eight native mutualist species, so $F_{1}$ is calculated to be equal to 4/8=0.5. For $F_{2}$, there are six links between the invasive species and ten native plant species, the value of $F_{2}$ is equal to 6/10=0.6.


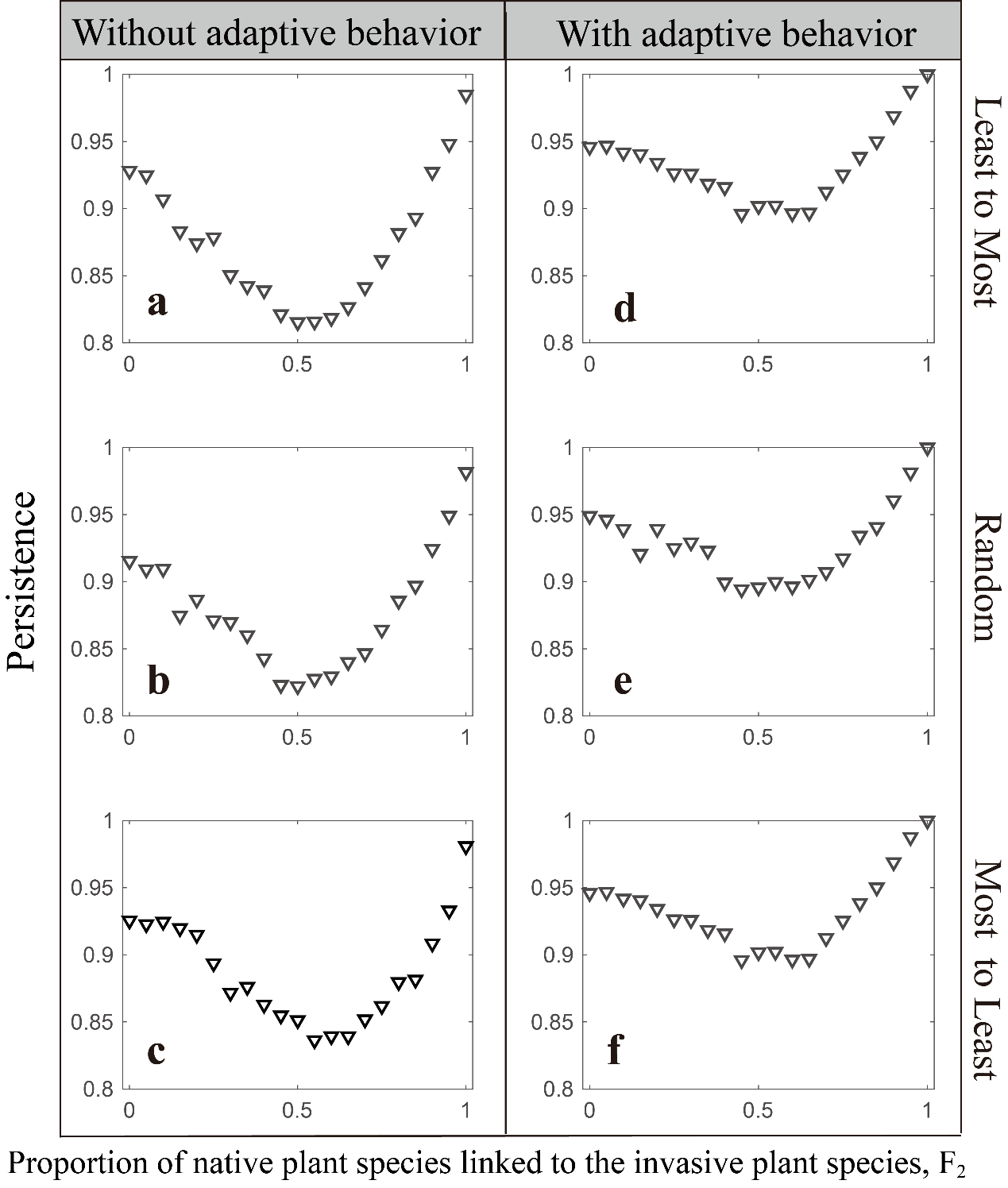


**Fig. S2.** Mean community persistence at steady state after invasion in mutualist-plant-herbivore multilayered networks with increasing $F_{2}$ (proportion of interaction links between invasive plant species and native plant species) at a fixed $F_{1}$ (proportion of interaction links between invasive plant species and native mutualist species). There are three linkage rules for invader linking to native (both plant and mutualist) species: Least-to-Most (**a** and **d**), Random (**b** and **e**) and Most-to-Least (**c** and **f**). (**a**, **b** and **c**) show invasive plant species without adaptive behavior. (**d**, **e** and **f**) are the scenarios with adaptive behavior.

Competition strength between invasive plant species *k* and native plant species *i* is simulated for two parameter settings, as described here. The first simulation used in the main text is: $\alpha_{ki}$ is drawn from a half-normal distribution $|N\left[ 0, \sigma^{2} \right]|$ with standard deviation ($\sigma$) drawn from $U[0.3, 0.5]$, and $\alpha_{ik}$ is drawn from same $|N\left[ 0, \sigma^{2} \right]|$ with $\sigma$ from $U[5, 5.5]$. This means that the probability $p\left( \alpha_{ki}<\alpha_{ik} \right)$ is higher than $p\left( \alpha_{ki}>\alpha_{ik} \right)$. The second simulation used in the supplementary materials is: $\alpha_{ki}$ and $\alpha_{ik}$ are independently drawn from the same half-normal distributions $|N\left[ 0, \sigma^{2} \right]|$ with $\sigma$ drawn from $U[0.3, 0.5]$. By using this method, the probability $p\left( \alpha_{ki}>\alpha_{ik} \right)$ is the same as $p\left( \alpha_{ki}<\alpha_{ik} \right)$. Comparing these two simulations can determine whether great difference in competitive ability affects community persistence and the relationship between native diversity and the probability of successful invasion.


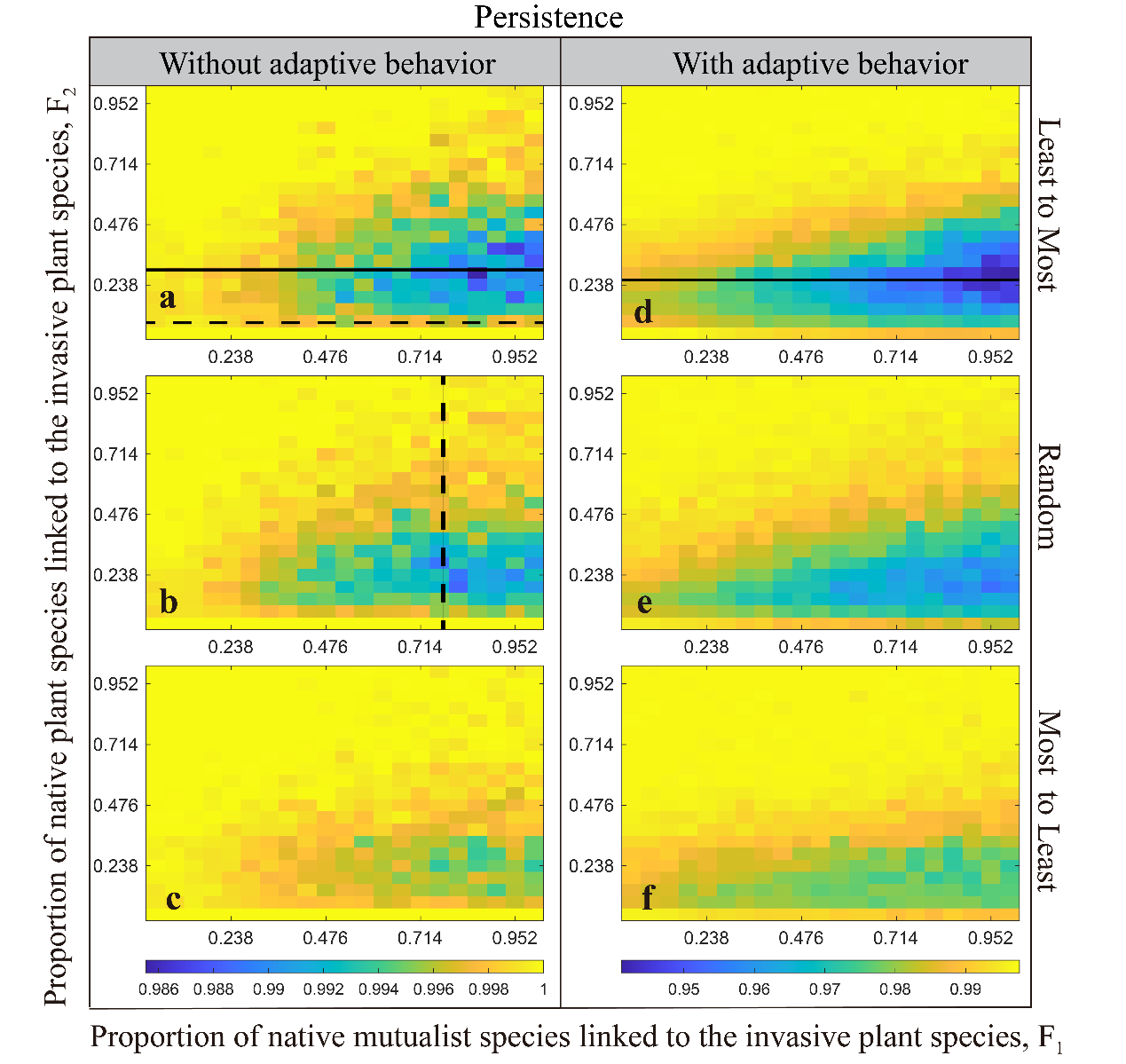


**Fig. S3.** Mean community persistence at steady state (averaged over 4 replicates) after invasion in mutualist-plant-herbivore multilayer networks. The proportions ($F_{1} \mathrm{and} F_{2}$) of native (mutualist or plant) species linked to the invader are varied ($0\leq F_{1}, F_{2}\leq1$). There are three linkage rules for invader linking to native (both plant and mutualist) species: Least-to-Most (**a** and **d**), Random (**b** and **e**) and Most-to-Least (**c** and **f**). (**a**, **b** and **c**) show invasive plant species without adaptive behavior. (**d**, **e** and **f**) are the scenarios with adaptive behavior.


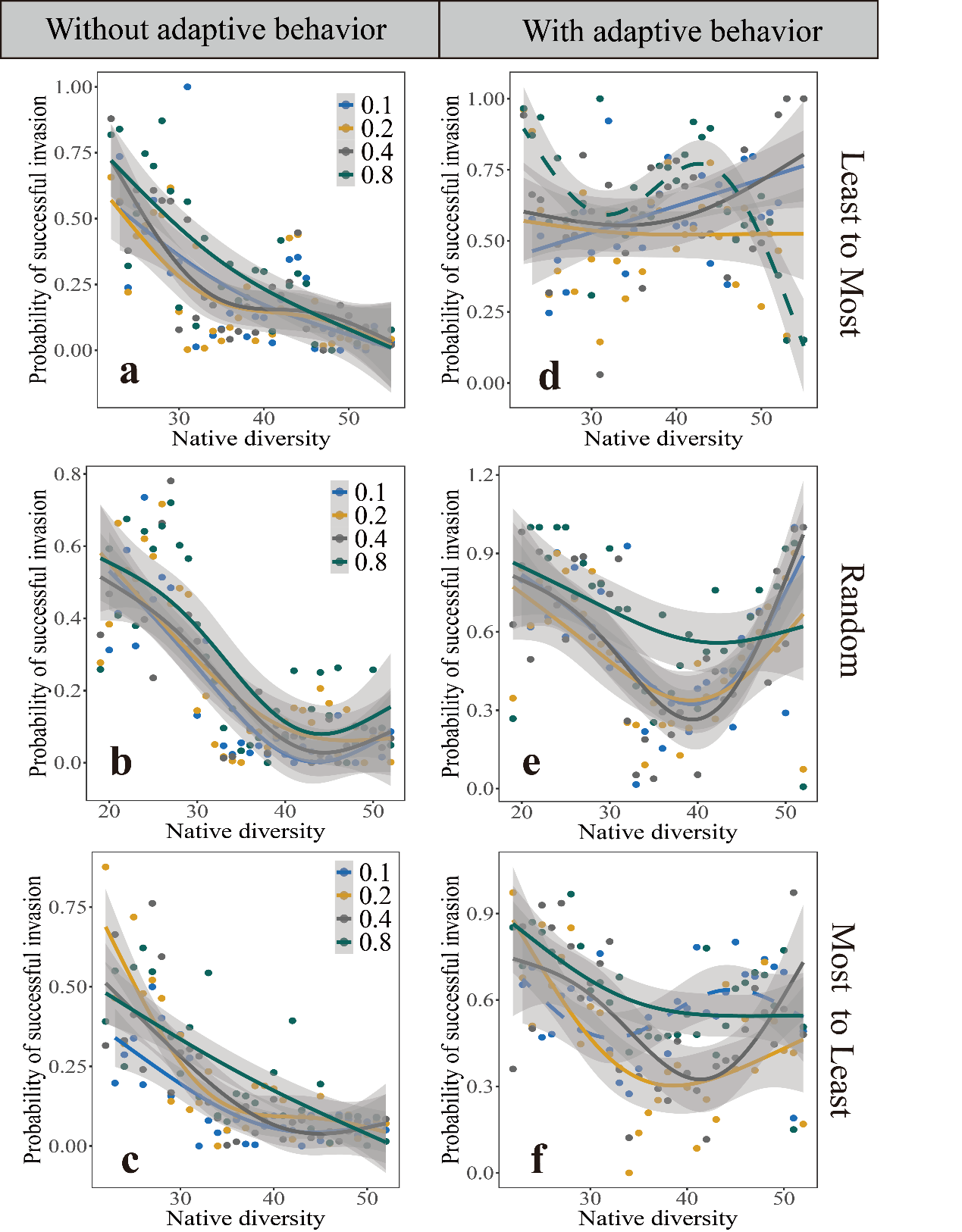


**Fig. S4.** The relationships between the probability of successful invasion and native diversity, evaluated at different proportions of native plant species linked to the invasive plant species ($F_{1}$ and $F_{2}$). Each column from top to bottom corresponds to Least-to-Most (**a** and **d**), Random (**b** and **e**) and Most-to-Least linkage rules (**c** and **f**), respectively. (**a**, **b** and **c**) are invasive plant species without adaptive behavior and (**d**, **e** and **f**) are with adaptive behavior. Colors indicate different proportions ($F_{2}$) of native plant species ($F_{2}=0.1, 0.2, 0.4, 0.8$) linked to the invasive plant species at a fixed proportion ($F_{1}$) of native mutualist species linked to the invasive plant species ($F_{1}=0.4$). Dashed curves indicate nonsignificant relationship at$P>0.05$ level. Native diversity refers to total species richness of plants, pollinators and herbivores.


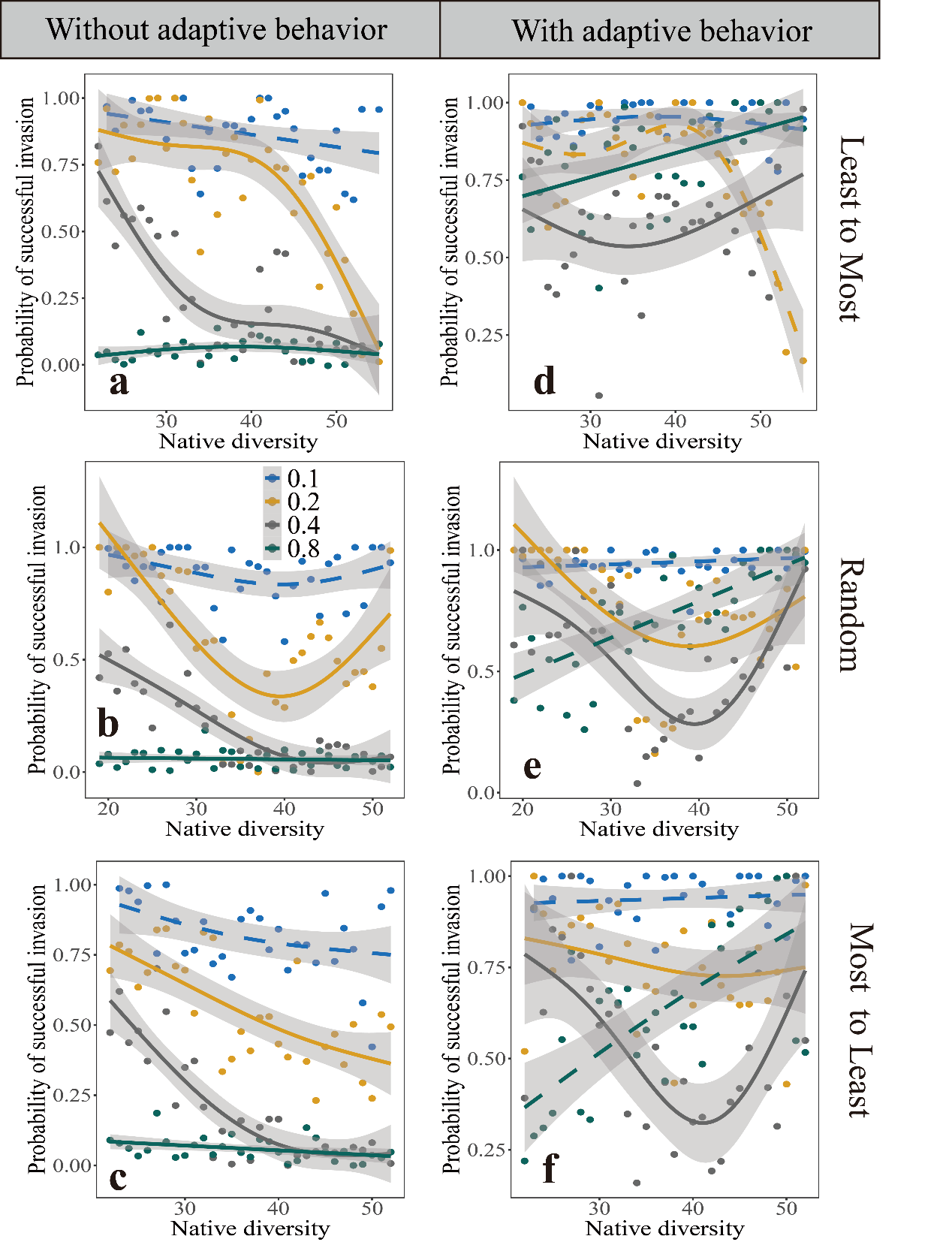


**Fig. S5.** The relationships between the probability of successful invasion and native diversity, evaluated at different proportions of native mutualist species linked to the invasive plant species. Each column from top to bottom corresponds to Least-to-Most (**a** and **d**), Random (**b** and **e**) and Most-to-Least linkage rules (**c** and **f**), respectively. (**a**, **b** and **c**) are invasive plant species without adaptive behavior and (**d**, **e** and **f**) are with adaptive behavior. Colors indicate different proportions of native mutualist species ($F_{1}=0.1, 0.2, 0.4, 0.8$) linked to the invasive plant species at a fixed value ($F_{2}=0.4$). Dashed lines indicate nonsignificant relationship at $P>0.05$ level. Native diversity refers to total species richness of plants, pollinators and herbivores.
